# Complex Residual Attention U-Net for Fast Ultrasound Imaging from a Single Plane-Wave Equivalent to Diverging Wave Imaging

**DOI:** 10.3390/s24165111

**Published:** 2024-08-07

**Authors:** Ahmed Bentaleb, Christophe Sintes, Pierre-Henri Conze, François Rousseau, Aziliz Guezou-Philippe, Chafiaa Hamitouche

**Affiliations:** Département Image et Traitement de l’Information, Institue Mines-Télécom (IMT) Atlantique, 29200 Brest, France

**Keywords:** complex convolutional neural networks, deep learning, image reconstruction, in-phase/quadrature signal, ultrasound imaging

## Abstract

Plane wave imaging persists as a focal point of research due to its high frame rate and low complexity. However, in spite of these advantages, its performance can be compromised by several factors such as noise, speckle, and artifacts that affect the image quality and resolution. In this paper, we propose an attention-based complex convolutional residual U-Net to reconstruct improved in-phase/quadrature complex data from a single insonification acquisition that matches diverging wave imaging. Our approach introduces an attention mechanism to the complex domain in conjunction with complex convolution to incorporate phase information and improve the image quality matching images obtained using coherent compounding imaging. To validate the effectiveness of this method, we trained our network on a simulated phased array dataset and evaluated it using in vitro and in vivo data. The experimental results show that our approach improved the ultrasound image quality by focusing the network’s attention on critical aspects of the complex data to identify and separate different regions of interest from background noise.

## 1. Introduction

Plane wave (PW) ultrasound (US) imaging [1] supports a high frame rate by transmitting a single unfocused beam that insonifies the field of view. This technique provides a high temporal resolution but limited lateral resolution and contrast. Multi-transmission of steered beams or diverging waves (DWs) [2] has successfully enhanced PW imaging; however, this technique is constrained by the number of transmitted waves, which directly affect the frame rate. This restricts its application in fast-moving structures such as the heart, and as such, PW imaging is the preferred method.

The transmitted waves (PWs or DWs) propagate through the target region and encounter impedance mismatches, causing an echo to reflect back to the transducer. In US image reconstruction, the reflected echo is received as a multichannel raw radio frequency (RF) signal. RF data are demodulated to produce in-phase/quadrature (IQ) data, followed by beamforming of the imaged target. Beamforming refers to the application of a time-of-flight correction process with spatial filtering to introduce selectivity into the signal, which eliminates undesirable interference [3].

Delay and sum (DAS) [4] is the most prevalent beamforming technique in medical US due to its low complexity and high frame rate. PW image beamforming with DAS results in a high frame rate but low image quality, whereas DW images are reconstructed from coherently compounding consecutive beamformed echos from each steered wave. Coherent compounding produces higher-quality images than PW imaging, but at the expense of a lower frame rate and a high computation cost related to the number of transmitted waves.

Deep learning has recently emerged as a critical component in the field of medical data analysis. Significant progress has been made in tasks such as image classification [5], segmentation [6,7], and liver and breast lesion classification [8,9], prompting US medical researchers to apply deep learning methods to their own work. Deep neural networks (DNNs) have been used for RF data interpolation [10] and reconstruction of B-mode images [11]. However, a more promising approach is the use of convolutional neural networks (CNNs), which been successfully applied to image processing-related tasks. For instance, CNNs have been used for compounding imaging [12], denoising, and speckle reduction [13], in addition to fully convolutional neural networks (FCNNs), which learned a minimum variance beamformer transformation [14] and direct image segmentation from RF data [15].

Most of the advancements achieved in the context of deep learning are based on real-valued data, whereas researchers have only recently begun to exploit the usability of complex-valued data. Complex-valued neural networks (CVNNs) were used in comparison with real-valued neural networks (RVNNs) [16] to identify the usability of such data and the learning of a complex representation of time series [17].

In US imaging, complex data comprise the raw demodulated IQ data from the RF signal and the complex DAS IQ data. As both contain phase and magnitude information, the latter are almost real (in other words, the module produces the US image); indeed, such data remain complex due to misknowledge with regard to the speed at which sound travels in human tissue.

A CNN was applied to complex data for speckle reduction [13] and improved multiline acquisition and transmission [18], where the complex data were trained as separate real and imaginary parts in a two-branch network structure. Consequently, such work failed to consider the nature of complex data. As demonstrated in [16], a complex-valued model provides a more constrained system than a model based on real-valued parameters. A recent study demonstrated the superior representational capacity of CVNNs in acoustic applications, such as speech spectrum prediction and music transcription [19]. A different approach was considered by [20,21]; in their work, a complex-valued CNN (CVCNN) was defined for fast US image reconstruction from DAS IQ data. The CVCNN consisted of a convolution between the complex data and complex weights represented as real and imaginary parts.

Current approaches to representing and processing IQ data in US imaging have used CNNs with complex convolutions without incorporating attention mechanisms. While an attention mechanism was applied to image despeckling in [22], it was limited to B-mode images and did not incorporate IQ data or complex data representations. To take advantage of the previously cited techniques for US image reconstruction, we propose a novel approach that combines the attention mechanism [23] with complex convolution based on a U-Net network [6] for US image reconstruction from a single PW acquisition. The reduction in transmitted waves will improve the frame rate, reduce the hardware complexity, and eventually improve the quality and resolution.

The aim of this work is to demonstrate the potential of CVNNs for IQ-based data in US image reconstruction tasks compared to image-based models using a simulated training dataset. While we will perform inference using in vivo samples, real-world data are different from simulations; therefore, the model requires further training and tuning using clinical data to be tested on real-world scenarios. In this work, we will address the following:

(1) A complex residual attention U-Net network (C-Res-Att-UNet) for US image reconstruction from a single PW matching DW imaging quality. This innovative framework leverages the phase information present in complex IQ data to enhance the representation, ultimately reconstructing higher-quality US images.
(2) A custom concatenation layer that takes into account complex data representation and a 2D max-pooling layer dedicated to down-sampling complex-valued data based on the indices of the maximum amplitude of the complex tensor.
(3) A complex up-sampling technique that learns an up-sampling transformation based on sub-pixel convolutions [24] rather than interpolating the real and imaginary parts separately.
(4) An equivalent network that is trained using a real-valued CNN named Res-Att-UNet, which uses B-mode image data.
(5) Evaluation of C-Res-Att-UNet and Res-Att-UNet using a test set from the simulated dataset, in addition to samples from the PICMUS dataset [25] containing in vitro phantom, simulation, and in vivo carotid data.

The present study is structured as follows: Section 2 describes the custom-built layers, the proposed architecture, and the training strategy. Section 3 presents the experimental setup for the data acquisition, network training, and performance metrics. Section 4 presents the results of the work. The discussion is presented in Section 5. Finally, the conclusion is outlined in Section 6.

## 2. Materials and Methods

### 2.1. Complex Convolution

This work focuses on incorporating the phase information present in complex IQ data into the attention mechanism to facilitate more effective learning of ultrasound data. We start by defining a complex data frame, $X=X_{r}+jX_{i}$ (where Xr=Re(X) and Xi=Im(X) are the real and imaginary components of *X*, respectively), and in the same manner, we describe a complex weight, $W=W_{r}+jW_{i}$. According to the definition provided in [19], the complex convolution *Z* of *X* with *W* is defined as follows:

(1) $$\begin{array}{cc} Z & =\left(X_{r}+jX_{i}\right)*\left(W_{r}+jW_{i}\right)\\ \; & =X_{r}*W_{r}-X_{i}*W_{i}+j\left(X_{r}*W_{i}+X_{i}*W_{r}\right), \end{array}$$

where (∗) represents the convolution operator. Rearranging and rewriting (1) (with *X* as the input and *Z* as the output) in a matrix format, we obtain the following:

(2) Re(Z)Im(Z)=Wr−WiWiWr∗XrXi.

In contrast to a purely real convolution in which the real and imaginary parts are considered to be part of a two-branch structure, in this paper, we construct a mathematical connection between the real and imaginary parts of the data frame and the convolution weights.

In order to update the weights after the forward pass, the condition for the back-propagation must be valid; i.e., the activation and loss functions must be differentiable. Sarroff et al. [17] stated that these functions must be holomorphic (complex differentiable functions that satisfy the Cauchy–Riemann conditions), whereas the authors of [16,19] showed that CVNNs can be optimized with real-valued activation and loss functions.

ReLU-based activation functions are commonly implemented in deep learning. We employ a complex ReLU (CReLU) activation function [19]. This choice is aligned with the differentiability condition described in [16]. (CReLU) is applied to the real and imaginary parts separately:

(3) CReLU(Z)=ReLU(Re(Z))+jReLU(Im(Z)).

Following [16,19], the network is evaluated at the end of each epoch using a positive real-valued loss function consisting of a mean squared error between the desired output image *Y* and network-reconstructed image $\hat{Y}$:

(4) $$L=\frac{1}{N}\sum^{N}{\left(Y-\hat{Y}\right)}^{2}.$$

### 2.2. Complex Concatenation Layer

A regular concatenation layer works by stacking the inputs one by one. This method cannot be applied to complex data, as the stacking would mix the real and imaginary parts. To overcome this problem, we implemented a complex concatenation layer (CConcat) that stacks the real components of the inputs followed by the imaginary components to achieve a correct stacking order, producing an output that is consistent with the definition of the complex convolution, (2). CConcat is defined as follows:

(5) Re(Zoutput)=Concat[Re(Z1),Re(Z2)]Im(Zoutput)=Concat[Im(Z1),Im(Z2)],

where $Z_{output}$ is the concatenated complex output from the complex inputs $Z_{1}$, and $Z_{2}$.

### 2.3. Complex Max-Pooling Layer

Usually, a pooling layer is used to reduce the spatial dimension of an input tensor across its features. The input is split into patches, and each patch is replaced by one value, which is defined by the pooling function. In max-pooling, the function is defined as the maximum value in the patch. Unfortunately, this could not be projected to the complex domain, where, in this work, the input tensor was defined as having real and imaginary components. The regular max-pooling layer’s output misplaces both real and imaginary components, resulting in the loss of the complex numbers (placing a real part in the same pool as a different imaginary part, or vice versa) and a consequent loss of phase information.

**Figure 1:**
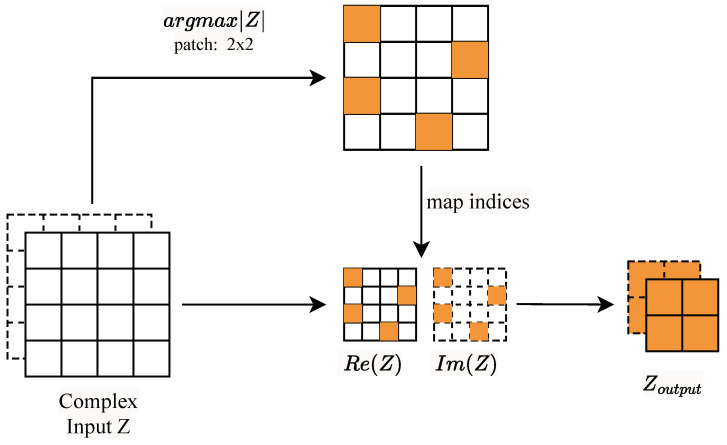
Block diagram of the proposed complex max-pooling layer. Solid and dashed lines indicate real and imaginary parts, respectively.

To overcome these issues, we suggest a complex max-pooling layer (CMax-pooling), presented in Figure 1. CMax-pooling is based on the maximum magnitude of the complex number in a patch. The CMax-pooling function is defined as follows:

(6) $$Z_{output}=Z\left(argmax\;|Z|\right),$$

where argmax |Z| is the index of the maximum magnitude used to extract the real and corresponding imaginary components. In this manner, the phase information is preserved by matching the correct components of the same complex number.

### 2.4. Complex Up-Sampling Layer

**Figure 2:**
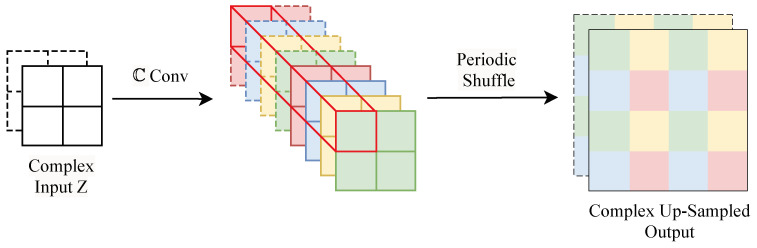
Block diagram of the proposed complex up-sampling layer with a factor of 2. The solid and dashed lines indicate real and imaginary parts, respectively.

The up-sampling layer, a ubiquitous component in many neural network architectures, typically employs interpolation techniques to resize input data to the desired dimensions. This technique might be successful if we resize the real and imaginary parts, separately. However, it fails to improve the reconstruction of the complex ultrasound signals. Our objective is to refine the reconstruction process at each step, which necessitates a more sophisticated approach. The proposed complex up-sampling layer (CUp-sampling) (Figure 2) is responsible for learning an up-sampling transformation based on periodic shuffling of the complex features from the complex convolution rather than relying on interpolation. This layer produces an up-sampled output that is not interpolated but rather learned from the rearranged complex features.

### 2.5. Proposed Network

**Figure 3:**
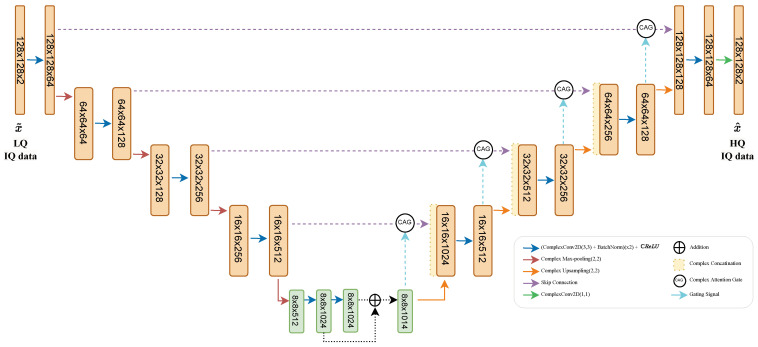
Block diagram of the proposed complex residual attention U-Net. $\tilde{x}$ is the low-quality (LQ) complex input and $\hat{x}$ is the high-quality (HQ) reconstruction.

The proposed network (Figure 3) contains two parts: an encoder and a decoder. The first part is composed of down-sampling blocks containing complex convolution layers, with batch normalization followed by CReLU activation (blue arrow). The down-sampling is performed using the custom CMax-pooling layer, where the tensor input is reduced using the index of the maximum magnitude across the max-pooling window to extract the corresponding real and imaginary parts. Prior to the implementation of the CMax-pooling layer (red arrow), the output is used as a skip connection (purple arrow). At the bottom of the encoder, a residual complex convolutional block is used in place of the traditional fully convolutional block.

The decoder part of the network is the area in which the HQ frame $\hat{x}$ is reconstructed based on information fed from the encoder. To reinforce the sensitivity and restoration accuracy, we employ an attention mechanism [23] in combination with the previously defined complex convolution (1), resulting in a complex attention gate (CAG) that combines the skip connection (purple arrow) from the encoder with information from the decoder CUp-sampling layers (cyan arrow). The skip connection provides more spatial information along with poor feature representation, while the attention gives more weight to the features of interest.

The features of CAG are defined as follows:

(7) $$CAG_{features}=W_{SG}\underset{c}{*}\left[CReLU\left(S\underset{c}{*}W_{S}+G\underset{c}{*}W_{G}\right)\right].$$

Meanwhile, the output of CAG is obtained as shown below:

(8) $$CAG_{output}=W_{CAG}\underset{c}{*}\left(S\times CAG_{features}\right),$$

where (×) represents a complex multiplication of *S*, a complex skip connection with $CAG_{features}$. *G* is the complex gating signal. $W_{\left(.\right)}$ is the complex kernel at each step, while $\left(\underset{c}{*}\right)$ represents the complex convolution defined in (2) between $W_{\left(.\right)}$ and different complex parameters along the CAG.

Instead of element-wise multiplication of the attention features with the skip connection signal, as defined in [23], a complex multiplication (8) is chosen, considering the nature of the presented complex data, followed by a complex convolution to enhance the output of the CAG and improve the overall learning process. The decoder is used symmetrically using up-sampling blocks with a CUp-sampling layer followed by a CConcat from the output of the CAG. It finishes with a complex convolution, leading to our HQ estimate $\tilde{x}$.

## 3. Experiment

### 3.1. Dataset Acquisition

**Figure 4:**
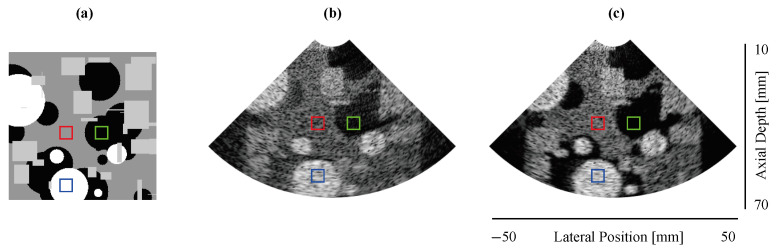
A sample from the phantom and training sets. The red area indicates a background region, the green area indicates an anechoic region, and the blue area indicates an hyperechoic region. (**a**) A computer-generated phantom used to simulate the dataset; (**b**) a B-mode image of low-quality IQ data acquired using single insonification, (**c**) a B-mode image of high-quality IQ data from standard compounding 20 steered acquisitions.

A phased-array image configuration based on a VERMON P2-8 transducer (VERMON SA, Tours, France) (number of elements: 80; center frequency: 2.8 MHz; element width: 200 μm; kerf: 40 μm) was used to simulate a training dataset, the open-source ultrasound simulator (SIMUS) [26,27], from computer-generated phantom images. The phantom images were constructed by randomly distributing sets of geometric shapes with varying dimensions and three levels of brightness over an image (Figure 4a). One phantom image is passed to SIMUS to generate the scatter points that are used for the US RF data simulation. Low-quality (LQ) input data were simulated using a single insonification acquisition (Figure 4b), whereas the high-quality (HQ) target data were obtained by coherently compounding 20 acquisitions that corresponded to the acquisition from steered angles between ±45° (Figure 4c). The acquired RF data were demodulated (via downmixing and low-pass filtering) and beamformed using DAS [4], with a mean velocity of 1540 m/s over a grid image of 128×128 pixels that corresponded to a depth of 60 mm (from 10 mm to 70 mm) and a sectorial angle of 90°. A total of 6000 phantom images were simulated to generate training pairs (LQ, HQ). We used the complex IQ data for the C-Res-att-UNet model and performed bmode conversion to obtain images for the Res-att-UNet model.

In addition to the simulated dataset, we evaluated both models using the PICMUS dataset, containing in vitro, simulated, and in vivo data. The PICMUS dataset was not included in the training.

### 3.2. Network Training

The network was implemented in Python 3.9 using a Keras API [28] and a Keras Complex API [29]. A selection of 4350 samples were used as the training set, 1100 were used for validation, and the remaining 550 samples were used for testing. The network was trained using an Adam optimizer [30] with a batch size of 7 and an initial learning rate of 0.001. An early stopping mechanism was implemented with a learning-rate scheduler to prevent over-fitting. The learning rate was halved if there was no reduction in the validation loss for 10 epochs, and the training was terminated if there were 25 epochs with no reductions in the validation loss. The training was conducted using a NVIDIA GeForce RTX 3080 GPU (Nvidia, Santa Clara, CA, USA).

### 3.3. Evaluation Metrics

To objectively assess the reconstruction performance of the proposed approach, we employ the following evaluation metrics:

Contrast-to-noise ratio (CNR) measures the signal–intensity ratio between the region of interest and the background [31]:

(9) $$CNR=\frac{\left|\mu_{R}-\mu_{B}\right|}{\sqrt{\sigma_{R}^{2}+\sigma_{B}^{2}}},$$

where $\mu_{R}$, $\mu_{B}$ ($\sigma_{R}$, $\sigma_{B}$) are the mean (standard deviation) of the region of interest and the background, respectively.

Structural similarity index (SSIM) measures the structural similarity between a given image and a reference image. The reference image is created via compounded imaging (20 DWs for the test set and 75 steered PWs for the PICMUS):

(10) $$SSIM=\frac{\left(2\mu_{\hat{Y}}\mu_{Y}+C_{1}\right)\left(2\sigma_{\hat{Y}Y}+C_{2}\right)}{\left(\mu_{\hat{Y}}^{2}+\mu_{Y}^{2}+C_{1}\right)\left(\sigma_{\hat{Y}}^{2}+\sigma_{Y}^{2}+C_{2}\right)},$$

where $\mu_{\hat{Y}}$ and $\mu_{Y}$ ($\sigma_{\hat{Y}}^{2}$ and $\sigma_{Y}^{2}$ ) are the means (variances) of $\hat{Y}$ and *Y*, respectively, $\sigma_{\hat{Y}Y}$ is the covariance between $\hat{Y}$ and *Y*, and $C_{1}$ and $C_{2}$ are two constants that stabilize the division with a weak denominator [21].

Peak signal-to-noise ratio (PSNR) is defined as the ratio of the image’s peak value to the distorting noise that degrades the quality of its representation [21]:

(11) $$PSNR=10\log_{10}\frac{MAX_{Y}^{2}}{RMSE[\hat{Y},Y]},$$

where $MAX_{Y}$ is the maximum pixel value of the image and RMSE is the root mean square error.

## 4. Results

### 4.1. Learning Convergence

The training ended according to the implemented early stopping mechanism for both networks, C-Res-Att-UNet and Res-Att-UNet, ensuring that the models reached an optimal state. The weights obtained from the last training session are used for further analysis. C-Res-Att-UNet required 133 epochs, with an average training time per epoch of 150 s. Res-Att-UNet, on the other hand, required 80 epochs, with an average training time per epoch of 38 s. Neither network suffered from under- or over-fitting. C-Res-Att-UNet required a longer training time, as we implemented custom layers that handle complex data and therefore necessitate more multiplication–addition operations.

### 4.2. Image Quality

The overall image quality obtained from both models was similar to that of the images obtained via standard compounding of 20 DWs. To assess the quality of both models, we performed inference using a test set and the PICMUS dataset (using simulated, in vitro, and in vivo data).

**Figure 5:**
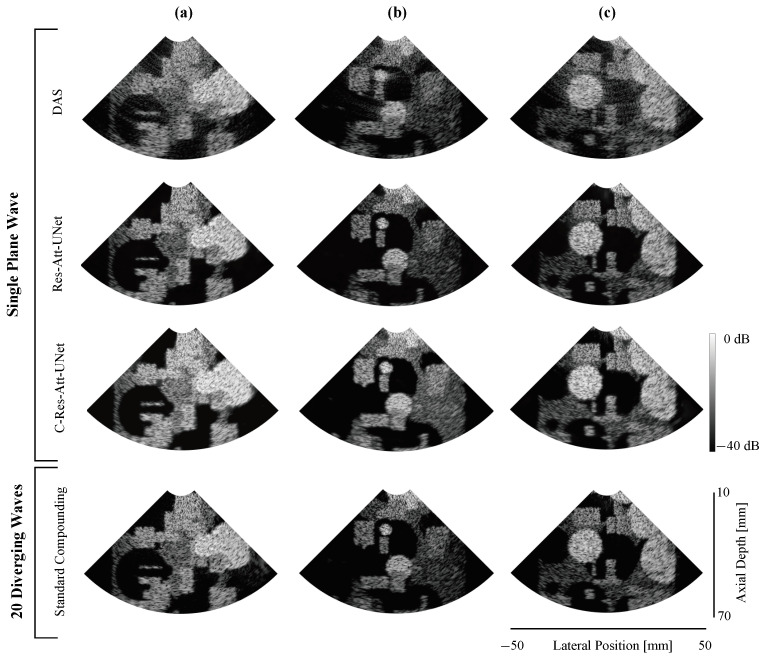
Three B-mode samples (**a**–**c**) from test datasets comparing different techniques. All results are from a single-plane wave insonification except for the standard compounding, which is obtained by coherently compounding 20 steered insonifications. Res-Att-UNet and C-Res-Att-UNet show visual improvements by separating different regions of noise.

**(1) Test set:** Figure 5 depicts samples from the test set displaying B-mode images of different techniques. C-Res-Att-UNet and Res-Att-UNet can produce images that are comparable to those created via DW imaging by effectively preserving important details and separating different regions from background noise. This improvement is attributed to the attention mechanism that is adapted to the complex domain in C-Res-Att-UNet and regular attention in Res-Att-UNet.

The previously defined evaluation metrics were computed over 40 samples from the test set for the three regions (background, anechoic, and hyperechoic), each represented by a 20×20 pixel square region (depicted in red, green, and blue in Figure 4). The results are presented in Table 1. We observe that C-Res-Att-UNet achieves the highest result in terms of CNR for the hyperechoic region, indicating superior contrast and visibility. For the anechoic region, Res-Att-UNet achieves the best result, while the reference of 20 DW achieves a lower result of same metric in the same region. Finally, comparing SSIM and PSNR using 20 DW images as the reference images, it is clear that Res-Att-UNet achieves the best PSNR value, while C-Res-Att-UNet presents a slightly better SSIM, indicating the closest similarity with the reference images. Overall, C-Res-Att-UNet achieves the most comparable performance to compounding imaging.

**Figure 6:**
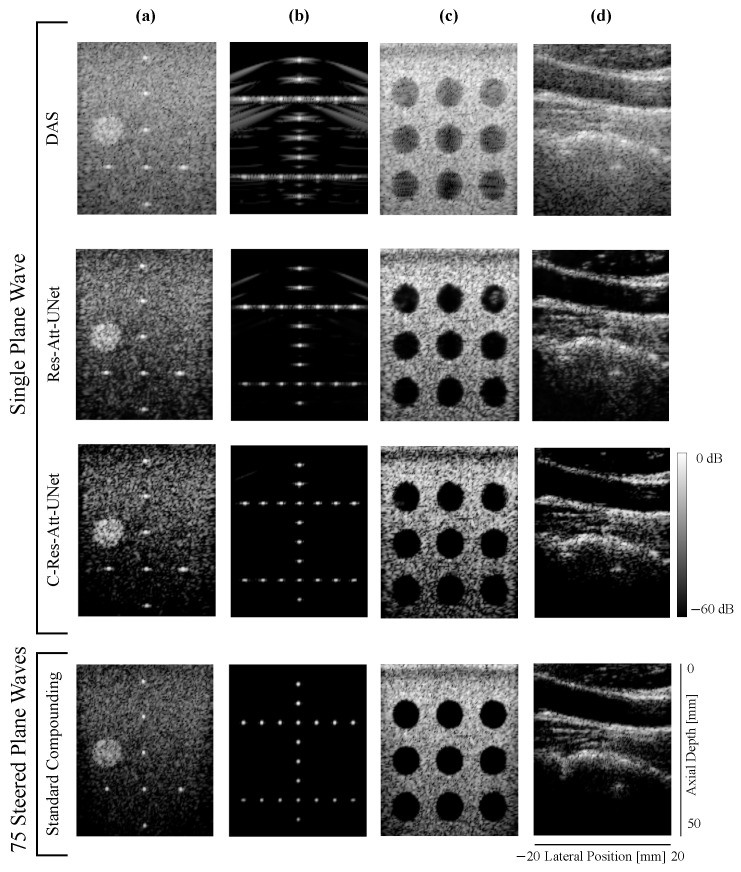
B-mode results of Res-Att-UNet and C-Res-Att-UNet with 1PW using the PICMUS dataset [25] compared to DAS (1PW) and standard compounding (75 PWS). (**a**) In vitro CIRS phantom, (**b**,**c**) simulated, and (**d**) in vivo carotid longitudinal section.

**(2) PICMUS:** Figure 6 illustrates the inference on the PICMUS dataset using the in vitro (CIRS phantom), simulated (point target and cyst target), and in vivo (carotid longitudinal section) data with a single PW. Both models successfully reconstructed images of similar visual quality in all four cases, offering a performance comparable to that of compounding imaging.

We computed the evaluation metrics for the image in Figure 6c for regions inside and outside the nine anechoic cysts. The results are presented in Table 2. Res-Att-UNet achieved superior results in terms of CNR, while C-Res-Att-UNet achieved the best results in terms of SSIM and PSNR, indicating the closest similarity with standard compounding of 75 PWs as a reference. Once again, C-Res-Att-UNet achieved the most comparable performance to compounding imaging.

Lastly, we computed the PSNR and SSIM metrics for the in vivo sample shown in Figure 6c. The Res-Att-UNet model achieved SSIM and PSNR values of 0.399 and 16.99, respectively. In comparison, the C-Res-Att-UNet model achieved higher values, with a SSIM of 0.595 and a PSNR of 18.54. Although both models showed improvements on the in vivo sample, they were not trained on real-world data. To ensure generalizability to clinical applications, further tuning using real-world data is necessary.

### 4.3. Lateral Resolution

**Figure 7:**
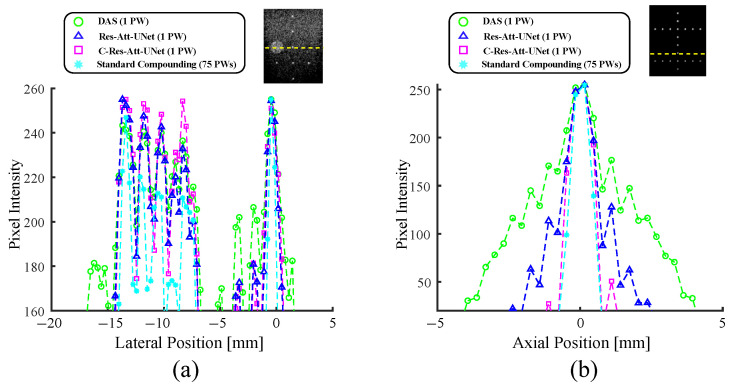
Lateral profiles of (**a**) the in vitro Figure 6 at a 26 mm depth and (**b**) the simulated point target Figure 6 at a 33 mm depth. Depth is indicated with a dashed yellow line.

The lateral profiles depicted in Figure 7 provide detailed insight into the performance of the trained models employing a single PW, the standard compounding of 75 PWs for in vitro data, and the point target represented in Figure 6. These profiles were taken at depths of 26 mm and 33 mm, respectively. In Figure 7, we observe that both models offers better reconstructions compared to single PW imaging (green circle). However, C-Res-Att-UNet (magenta square) demonstrates a high-quality reconstruction that is comparable to the target image (cyan asterisk) in both scenarios. The sharp reconstruction is related to the defined complex attention with the enhanced complex up-sampling layer.

### 4.4. Computational Performance

Table 3 presents the computational data of each model in terms of the number of training parameters, training time, and inference time on the GPU (inference was performed on the machine described in Section 3.2). Res-Att-UNet took around 50 min to train, whereas C-Res-Att-Unet took approximately 5.5 h to train. This indicates that a model that learns image-to-image mapping is significantly faster than a model attempting to learn complex-to-complex data mapping. This difference in training time has also been attributed to the built layers. For example, the concatenation layer for image data takes one concatenation step, while complex data take two concatenation steps for the real and imaginary parts and one last concatenation to produce a tensor that is compatible with a Keras Complex API. Another observation is the inference time on the GPU, which took 6 ms and 2 ms for C-Res-Att-UNet and Res-Att-UNet, respectively. We also compared the processing time of the PICMUS data with the provided scripts that ran on the CPU using MATLAB R2020a. The single PW took 1.02 s, while compounded imaging of 75 PWs took 42.84 s. Consequently, the marginal increase of 6 ms achieved by C-Res-Att-UNet remains unnoticeable with regard to compounded imaging, especially when considering the image quality achieved using a single PW.

## 5. Discussion

In this study, we presented a detailed analysis of a complex-valued UNet-based model for US image reconstruction from complex IQ signals in comparison to a standard DAS and UNet-based image model using a single PW. The results indicate that both Res-Att-UNet and C-Res-Att-UNet significantly improved the final US image, which was proved by the CNR, SSIM, and PSNR metrics compared to DAS. Specifically, C-Res-Att-UNet achieved the highest CNR in the hyperechoic region and the best SSIM, indicating the closest similarity to the standard compounding reference images in the simulated and PICMUS data.

In addition to the quantitative evaluation, a lateral resolution analysis was conducted at different depths (26 mm and 33 mm) for in vitro and simulated point target scenarios. The lateral profiles illustrate that both Res-Att-UNet and C-Res-Att-UNet offer superior image reconstruction quality compared to single PW imaging. Notably, C-Res-Att-UNet demonstrated a high-quality reconstruction that was comparable to the target image, attributed to its complex attention mechanism and enhanced complex up-sampling layer.

In summary, the analysis demonstrated that both Res-Att-UNet and C-Res-Att-UNet significantly enhanced US imaging quality from a single PW, achieving results close to those of standard compounding with multiple DWs. C-Res-Att-UNet consistently provided the highest SSIM and PSNR values, indicating its superior capability to preserve image structure and detail, making it a promising approach for fast and high-quality US imaging.

The downside of C-Res-Att-UNet was observed in the training time, as the network configuration doubled the number of addition–multiplication operations, leading to a slow learning time and relatively acceptable inference time concerning US imaging applications.

For future work, we would like to obtain real-world clinical data to improve and fine-tune the complex model to further investigate the performance of such an approach.

## 6. Conclusions

In this paper, we proposed a complex residual attention U-Net to achieve ultrasound image reconstruction using a single PW. The network uses complex convolution and attention mechanisms to incorporate phase information into training. We introduced additional layers to enhance complex data representation. An equivalent network of C-Res-Att-UNet named Res-Att-UNet for image data was trained for comparison. Our results showcase the performance of our approach to reconstructing images that are of a similar quality and resolution to compounded imaging in comparison to Res-Att-UNet. This improvement is attributed to the use of phase information and complex attention. However, our technique has several limitations: CVNNs require additional computation compared to RVNNs, which increases the training and inference time. Hence, further optimization is required. Nonetheless, this work will facilitate further investigations in complex data processing for a full CVCNN beamformer network, bypassing the DAS beamformer. 

## Figures and Tables

**Table 1:** CNR, SSIM, and PSNR of DAS (1PW), Res-Att-UNet (1 PW), C-Res-Att-UNet (1 PW), and standard compounding (20 DWs).

Method	Hyperechoic Region CNR	Anechoic Region CNR	SSIM	PSNR
DAS (1 PW)	1.183	1.061	0.227	14.35
Res-Att-UNet (1 PW)	1.278	**1.012**	0.825	**23.86**
C-Res-Att-UNet (1 PW)	**1.314**	0.994	**0.836**	21.74
Standard compounding (20 DWs)	1.220	0.940	1	∞

Bold highlights the best value for each metric across different methods.

**Table 2:** CNR, SSIM, and PSNR of DAS (1 PW), Res-Att-UNet (1 PW), C-Res-Att-UNet (1 PW), and standard compounding (75 PWs) of Figure 6c.

Method	CNR	SSIM	PSNR
DAS (1 PW)	1.120	0.489	11.316
Res-Att-UNet (1 PW)	**1.157**	0.673	17.446
C-Res-Att-UNet (1 PW)	0.919	**0.718**	**17.611**
Standard compounding (75 DWs)	1.0241	1	∞

Bold highlights the best value for each metric across different methods.

**Table 3:** Number of parameters, training time, and inference time of Res-Att-UNet and C-Res-Att-UNet.

Model	Number of Parameters	Training Time	Inference Time (GPU)
Res-Att-UNet	29 million	50 min	2 ms
C-Res-Att-UNet	31 million	5.5 h	6 ms

## Data Availability

The data can be made available from the corresponding author upon reasonable request.
